# Seasonal polyphenism of spotted‐wing *Drosophila* is affected by variation in local abiotic conditions within its invaded range, likely influencing survival and regional population dynamics

**DOI:** 10.1002/ece3.6491

**Published:** 2020-06-24

**Authors:** Dara G. Stockton, Anna K. Wallingford, Gabrielle Brind'amore, Lauren Diepenbrock, Hannah Burrack, Heather Leach, Rufus Isaacs, Lindsy E. Iglesias, Oscar Liburd, Francis Drummond, Elissa Ballman, Christelle Guedot, Janet Van Zoeren, Greg M. Loeb

**Affiliations:** ^1^ Department of Entomology Cornell AgriTech Cornell University Geneva New York USA; ^2^ University of New Hampshire Cooperative Extension Durham New Hampshire USA; ^3^ Department of Entomology and Nematology University of Florida Lake Alfred Florida USA; ^4^ Department of Entomology and Plant Pathology North Carolina State University Raleigh North Carolina USA; ^5^ Department of Entomology The Pennsylvania State University University Park Pennsylvania USA; ^6^ Department of Entomology Michigan State University East Lansing Michigan USA; ^7^ Department of Entomology and Nematology University of Florida Gainesville Florida USA; ^8^ School of Biology and Ecology University of Maine Orono Maine USA; ^9^ Cooperative Extension University of Maine Orono Maine USA; ^10^ Department of Entomology University of Wisconsin Madison Wisconsin USA

**Keywords:** cold tolerance, *Drosophila*, overwintering, phenotypic plasticity, polymorphism, SWD, winter morph

## Abstract

1. Overwintering *Drosophila* often display adaptive phenotypic differences beneficial for survival at low temperatures. However, it is unclear which morphological traits are the best estimators of abiotic conditions, how those traits are correlated with functional outcomes in cold tolerance, and whether there are regional differences in trait expression.
2. We used a combination of controlled laboratory assays, and collaborative field collections of invasive *Drosophila suzukii* in different areas of the United States, to study the factors affecting phenotype variability of this temperate fruit pest now found globally.
3. Laboratory studies demonstrated that winter morph (WM) trait expression is continuous within the developmental temperature niche of this species (10–25°C) and that wing length and abdominal melanization are the best predictors of the larval abiotic environment.
4. However, the duration and timing of cold exposure also produced significant variation in development time, morphology, and survival at cold temperatures. During a stress test assay conducted at −5°C, although cold tolerance was greater among WM flies, long‐term exposure to cold temperatures as adults significantly improved summer morph (SM) survival, indicating that these traits are not controlled by a single mechanism.
5. Among wild *D. suzukii* populations, we found that regional variation in abiotic conditions differentially affects the expression of morphological traits, although further research is needed to determine whether these differences are genetic or environmental in origin and whether thermal susceptibility thresholds differ among populations within its invaded range.

## Graphical abstract

We explored how seasonal traits are correlated with functional outcomes in cold tolerance in *D. suzukii*, and whether there are regional differences in trait expression. While wing length and abdominal melanization were the best predictors of the larval abiotic environment, the duration and timing of cold exposure also produced significant physiological variation. Because we observed morphotype variation among wild *D. suzukii* populations, in different regions of the Eastern United States, a population‐centered research and modeling approach may be required to assign morphotype labels and predict overwintering survival outcomes.

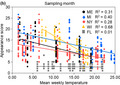

## 1 INTRODUCTION

Phenotypic plasticity allows organisms within a given genotype to respond adaptively to the challenges posed by environmental variability via beneficial shifts in morphology, physiology, or behavior (Agrawal, 2001; Thompson, 1993; West‐Eberhard, 1989). The resulting changes are known to broadly affect patterns of dispersal, diet use, and reproduction and are well documented in a wide diversity of species, most notably arthropods (Fusco & Minelli, 2010; Heidinger, Hein, & Bonte, 2010; Whitman & Agrawal, 2009). Indeed, the propensity for phenotypic variation and plasticity in trait expression among arthropods is considered a key reason for their widespread success and diversity, even in extreme climates (Moczek, 2010; Nijhout, 1999; Pfennig et al., 2010; West‐Eberhard, 1989). Seasonal polyphenism, the predictable shift in phenotype expression associated with temporal changes in the environment, is common among overwintering species which require the ability to shift from a foraging/reproductive phase, to one of survival and metabolic dormancy (Hodkinson, Bird, Miles, Bale, & Lennon, 1999; Shapiro, 1976; Sinclair, 1999). The biochemical mechanisms associated with seasonal trait expression are often induced by specific abiotic thresholds (e.g., temperature, photoperiod, state of hydration) early in development and prepare the individual for thermal stress tolerance through changes in carbohydrate metabolism, dietary cryoprotectant sequestration, or the creation of ice‐nucleation proteins (Baust, 1981; Ohtsu, Kimura, & Katagiri, 1998; Sinclair, 1999; Strachan, Tarnowski‐Garner, Marshall, & Sinclair, 2011). In addition, the external morphology of cold tolerant arthropods often undergoes change as well (Bale, Hansen, & Baust, 1989; Kimura, Awasaki, Ohtsu, & Shimada, 1992; Storey & Storey, 1986). In cool environments, arthropod larvae generally take longer to complete development than those of the same species reared at warmer temperatures (Holloway, Marriot, & Crocker, 1997; Kimura, 1988; Nyamaukondiwa, Terblanche, Marshall, & Sinclair, 2011). Subsequently, those adults are larger and display darker cuticular melanization than those individuals reared at warmer temperatures, traits which are thought to help retain heat (Atkinson & Sibly, 1997; Kingsolver & Wiernasz, 1991; Shearer et al., 2016; Wallingford & Loeb, 2016). Insects displaying these differentially expressed traits are often referred to as winter morphs (WM) or winter‐form insects (David et al., 1994; Oldfield, 1970; Pétavy, Moreteau, Gibert, & David, 2002), and are prevalent in cool temperate climates, where organisms have evolved strategies to cope with harsh winter conditions (Danks, 2004; Shapiro, 1976; Strathdee & Bale, 1998; Tauber & Tauber, 1981). Indeed, in addition to predictable, cyclic changes in phenotype expression, there is growing evidence of genetic changes on a population level among some species due to changing climate (Hoffmann & Sgró, 2011; Somero, 2010). This is particularly significant in the case of invasive species because thermal limits and the capacity to adapt to novel environments directly affects the potential geographic distribution and thus, the risk of economic damage associated with an expanding host range (Paini et al., 2016; Terblanche, Deere, Clusella‐Trullas, Janion, & Chown, 2007).

Phenotypic variation is well documented among *Drosophila,* and when reared at cooler temperatures, genetic selection for adults with larger body size occurs within a few generations (Ayrinhac et al., 2004; Hoffmann & Hercus, 2000; Hoffmann, Sorensen, & Loeschchke, 2003; Neat, Fowler, French, & Partridge, 1995; Rako & Hoffmann, 2006). This suggests that the ability to survive novel climates may be heritable within *Drosophila* populations over time (Hoffmann et al., 2003). Genetic analysis of *Drosophila melanogaster* has shown that loci associated with wing shape and size are directly affected by thermal selection and that wing morphology has adaptive significance in relation to temperature (Cavicchi, Giorgi, Natali, & Guerra, 1991). This is likely because large wings are more effective at heat absorption, making them advantageous during cool conditions when heat acquisition and retention are critical (Douglas, 1981; Heinrich, 1974; Kingsolver & Koehl, 1985). While this is fundamentally a byproduct of slowed development on an individual level, there may also be population‐level effects selecting for improved survival under cool conditions (Gotthard, Nylin, & Nylin, 1995; Hoffmann & Hercus, 2000; Hoffmann et al., 2003; Overgaard, Kristensen, Mitchell, & Hoffmann, 2011). In this case, a species would be said to have acquired some measure of genetic adaptation in response to selection events, rather than merely an adaptive, plastic response to acute environmental conditions (Gotthard & Nylin, 1995).

There appears to be precedent for both events broadly among *Drosophila*. Some species such as *Drosophila bizonata* and *Drosophila daruma* display distinct strain variations in thermal tolerance despite when reared under similar conditions in the laboratory (Kimura, 2004). Among these species, restricted gene flow between allopatric populations in cool and warm climates has been suggested as a likely mechanism driving these genetic changes (Kimura, 2004). In contrast, little to no intraspecific variation in climatic adaptation has been observed among species in the melanogaster species group despite a wide geographic range (Kimura, 1988). This suggests some *Drosophila* species instead possess a more acute mechanism for phenotypic shifts (Kimura, 2004). Indeed, some estimates suggest as much as 80% of the variation in cold tolerance among *D. melanogaster* can be attributed to changes in phenotypic expression (Ayrinhac et al., 2004).

Spotted‐wing drosophila, *Drosophila suzukii* Matsumura (Diptera: Drosophilidae), is an invasive pest species that displays remarkable capacity for range expansion and local adaptation to extreme environmental conditions (Asplen et al., 2015; Stephens, Asplen, Hutchison, & Venette, 2015). Since its accidental introduction in California during 2009, this unique niche specialist (Stockton, Brown, et al., 2019; Stockton, Wallingford, et al., 2019) has moved rapidly across the continent, resulting in millions of dollars in economic losses in berry production (Bolda, Goodhue, & Zalom, 2010; Farnsworth et al., 2017; Walsh et al., 2011). The global range of *D. suzukii* now extends from its native habitat in East Asia, to central Asia, Europe, and both American continents, making it one of the most significant, and adaptable invasive species of the 21st century (Asplen et al., 2015; Deprá, Poppe, Schmitz, De Toni, & Valente, 2014; Gutierrez, Ponti, & Dalton, 2016; dos Santos et al., 2017). As a result, applied research over the last decade has greatly expanded our understanding of *D. suzukii* biology and ecology, including the influence of climatic conditions on overwintering success in its new geographic ranges (Dalton et al., 2011; Guédot, Avanesyan, & Hietala‐Henschell, 2018; Jakobs, Gariepy, & Sinclair, 2015; Leach, Stone, Van Timmeren, & Isaacs, 2019; Leach, Van Timmeren, Wetzel, & Isaacs, 2019; Panel et al., 2018; Rossi‐Stacconi et al., 2016; Stockton, Brown, et al., 2019; Stockton, Wallingford, & Loeb, 2018; Stockton, Wallingford, et al., 2019; Tochen et al., 2014; Toxopeus, Jakobs, Ferguson, Gariepy, & Sinclair, 2016; Tran, Hutchison, & Asplen, 2020; Wallingford & Loeb, 2016; Zerulla, Schmidt, Streitberger, Zebitz, & Zelger, 2015). Like other temperate *Drosophila*, *D. suzukii* expresses WM traits when reared at cool temperatures (Shearer et al., 2016) and appears capable of overwintering locally, even in regions with freezing temperatures for several months of the year (Rossi‐Stacconi et al., 2016; Rota‐Stabelli et al., 2020; Stockton, Brown, et al., 2019; Stockton et al., 2018; Stockton, Wallingford, et al., 2019; Tait et al., 2018). However, it remains unclear how abiotic conditions affect ontogenetic development, which is particularly important among species displaying multiple seasonal body forms (de Aranzamendi, Martínez, & Sahade, 2010). Studies in Oregon and Michigan have reported WM trait expression using the L4 longitudinal wing vein and found that wing size increases with decreasing temperature both in wild‐type and colony populations (Leach, Stone, et al., 2019; Shearer et al., 2016). Using a 0–5 rating scale (5 = darker), differences in seasonal abdominal melanization have also been reported with sex‐specific differences on 4th abdominal segment among WM females and on the 3rd segment in WM males (Shearer et al., 2016). Most recently, regression tree analysis has been used to estimate WM cutoff values for wild *D. suzukii* collected throughout the year during 2017–2018 in Minnesota (Tran et al., 2020). The authors of that study reported specific WM threshold values for wing length (greater than 2.69 mm) and wing: hind‐tibia ratio (greater than 2.17) in female specimens, although the measurements used were not consistent with the morphometric criteria used by other groups, nor did they include a metric of abdominal melanization, making comparisons with previous studies difficult.

Despite these advances, more information is needed to determine the influence of temperature on morphotype expression on a more continuous scale, and it remains unclear how these changes in morphology relate to winter stress tolerance. Given the overlap in morphotype expression observed by Leach, Stone, et al. (2019), the time of year in which *D. suzukii* develops may influence not only morphology, but also the relative degree of cold tolerance. Among other cool‐temperate *Drosophila* found in the native range of *D. suzukii*, the timing of seasonal development and the duration of exposure to cool temperatures is directly linked to triglyceride accumulation and overwintering survival (Ohtsu, Kimura, & Hori, 1995). Furthermore, previous research on the mechanisms underlying thermal acclimation in *D. suzukii* indicates that regulation of external morphology and internal physiology may not be directly linked. Indeed, our previous research has showed that even SM flies can develop cold tolerance if exposed to cool temperatures, as additional functional traits develop during the adult life stage (Stockton et al., 2018). For this reason, it is important that we understand how morphological trait expression and cold tolerance compare among *D. suzukii* whose exposure to cool temperatures begin early or late in larval development. Lastly, it is unclear whether morphotype expression is variable among regional populations, such as those collected from the northeastern versus southeastern United States. Currently, most field‐based research using wild specimens collected in the United States has focused on local populations in a single state or region (Guédot et al., 2018; Leach, Stone, et al., 2019; Shearer et al., 2016; Tran et al., 2020). While genetic analysis has found little difference in *D. suzukii* populations occupying climatically different regions of the United States (e.g., New York and North Carolina), well‐defined genetic clusters in the Eastern and Western sides of the country indicate limited movement following establishment (Fraimout et al., 2017). Distinct populations also exist between North America and Europe, likely due to similarly isolated invasion events and little secondary trans‐Atlantic movement (Rota‐Stabelli et al., 2020). Furthermore, at least some significant phenotype differences between populations have been identified, including changes in maternal fecundity, susceptibility to parasitoids, and Wolbachia frequencies (Rota‐Stabelli et al., 2020). These and other nongenetic differences require investigation using behavioral and physiological bioassays and are not likely to be identified by genetic analysis alone.

In this study, we aimed to determine the relationship between adaptive plasticity and thermal tolerance in *D. suzukii*. First, we developed a method for creating and characterizing adult WM flies using controlled bioassays to obtain morphometric measurements of wing, thorax, tibia size, and abdominal melanization to determine which traits were most strongly associated with changes in the larval abiotic environment. Next, we observed how the duration and timing of cold exposure during development affected both WM trait expression and thermal susceptibility. This was important because while internal and external traits associated with cold tolerance often develop concomitantly, expression may vary depending on the life stage at which cold exposure occurs (Stockton et al., 2018). Finally, we measured wild *D. suzukii* collected throughout the year in Michigan (MI), Wisconsin (WI), New York (NY), Maine (ME), and Florida (FL) during 2015–2018. These flies were analyzed to determine the degree of morphotype variation within and among populations in the Eastern United States, as this is fundamental to how we understand differential sources of genetic versus environmental variation (Gotthard et al., 1995; Hoffmann et al., 2003; Overgaard et al., 2011). If morphotype variation among specimens varies in a manner inconsistent with the abiotic conditions, this would suggest that these populations are genetically distinct (Ayrinhac et al., 2004; Hoffmann et al., 2003). By focusing on the factors affecting morphotype and cold tolerance variation among *D. suzukii* in both laboratory and field‐collected samples, we aim to better understand the relative thermal limits of survival in different regions of the invaded range. If significant regional differences in pest phenotype and cold tolerance are detected, a more population‐centered approach to future research and management of *D. suzukii* may be warranted (Reichard et al., 2015; Rota‐Stabelli et al., 2020).

## 2 MATERIALS AND METHODS

### 2.1 Fly colonies

Laboratory experiments used *D. suzukii* from two locations. One colony was housed at Cornell AgriTech Small Fruit Entomology in Geneva, New York (NY). It was sourced from wild *D. suzukii* collected locally from infested blueberry and raspberry fields during 2014, although new genetic material from these sites was added to the colony annually. The second *D. suzukii* colony was housed at North Carolina State University, Department of Entomology in Raleigh, North Carolina (NC). It originated from flies collected in 2010 at the Upper Mountain Research Station, Laurel Springs, NC, and was also refreshed annually with new genetic material from locations throughout NC. Both colonies were reared continuously from the time they began. Total population size of each colony varied yearly based on the experimental needs at the time, fluctuating between 2,000–10,000 flies per generation.

Similar environmental conditions and rearing practices were used at both locations for the purposes of this experiment. The flies were housed in 236 ml polypropylene rearing bottles (8 ounce Drosophila stock bottles; VWR International, Radnor, PA) containing 40 ml standard cornmeal‐agar *D. suzukii* diet including a methylparaben anti‐fungal additive (see Stockton, Brown, et al., 2019; Stockton, Wallingford, et al., 2019). Approximately 100 mixed‐sex flies were housed in each bottle, which was replaced once weekly until all adults died or were used in the study. Newly eclosed offspring flies were moved to new bottles to separate the flies by age. The SM colony environmental conditions were set at 25°C with a 16L:8D (light/dark photoperiodic cycle) at 55% relative humidity (RH). Unless otherwise stated, WM induction began 24 hr after oviposition by moving bottles of eggs (collected from the SM colony) to a 15°C growth chamber with a 12L:12D light cycle. After eclosion, we maintained the WM flies at 15°C until they were used for experiments.

### 2.2 Defining winter morph traits

**FIGURE 1:**
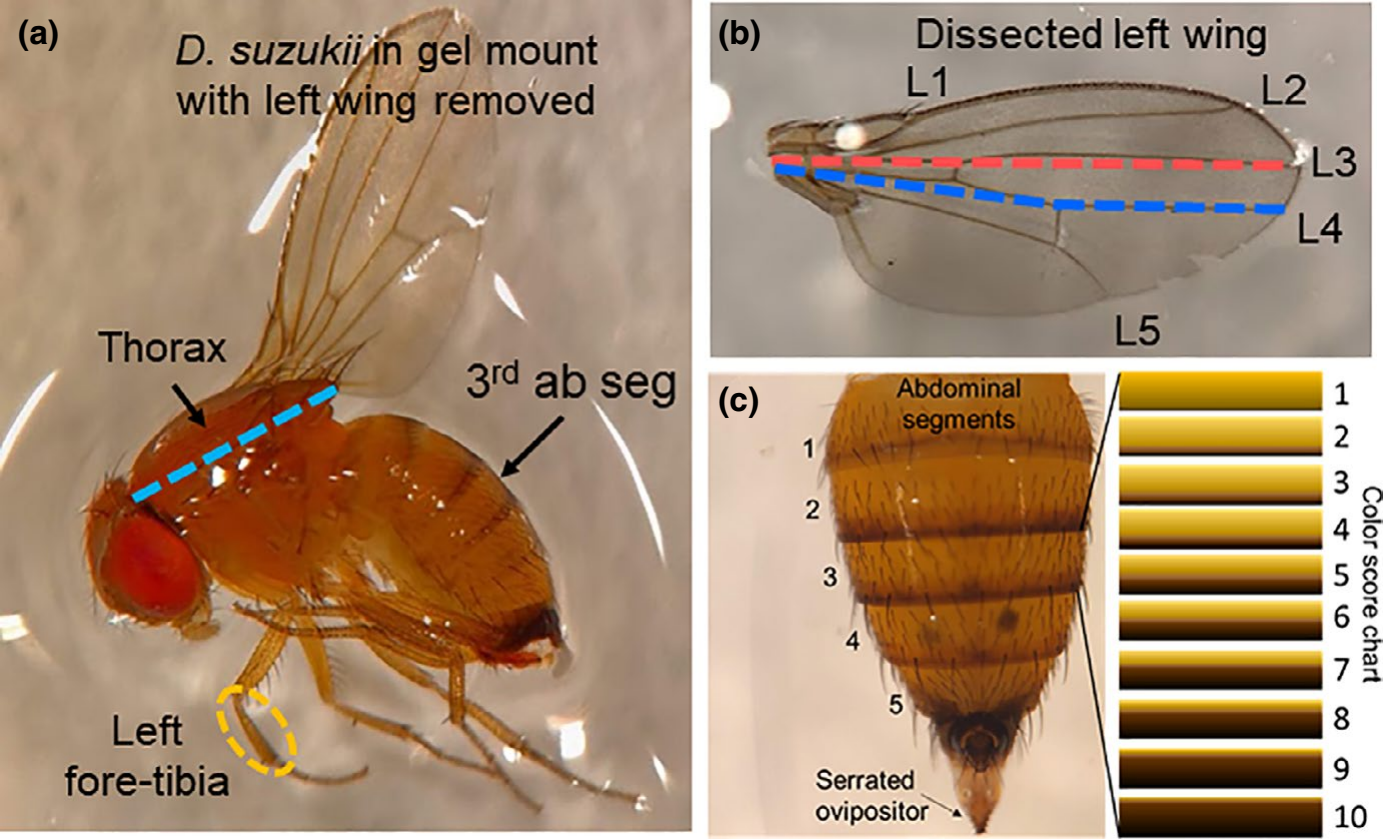
Morphometric characters assessed included *Drosophila suzukii* wing, thorax, and tibia length (a). The length of the L3 and L4 longitudinal wing veins was measured along the dissected left wing of each fly (b). Abdominal color score (1–10) was based on the percent melanization of the anterior dorsal abdominal tergites (c)

We assessed changes in wing length, thorax length, tibia length, and abdominal color score in SM and WM flies from NY (*N* = 32) and NC (*N* = 40). Equal numbers of males and females were measured from each state. Five days posteclosion, flies from either location were euthanized in 95% ethanol and stored at −4°C until dissection. Flies from NC were shipped to NY for evaluation. Morphometric assessments were conducted using a stereo microscope set at 10× magnification (Zeiss Stemi 508; Carl Zeiss Microscopy, LLC) with an attached digital interface (Moticam 5+; Motic America) and associated measuring software (Motic Images Plus 3.0). To standardize the positioning of the body (Figure S1), each fly was mounted in clear hand sanitizer gel (Purell hand sanitizer, GOGO Industries, Inc.), with the fly left‐side up (Figure 1a).

The left wing was dissected to obtain accurate wing length measurements and make the other body features more accessible. Two wing measurements were taken to compare how well each predicted WM body forms (Figure 1b). The first wing length measure was taken along the L3 longitudinal wing vein from the proximal end at the base of the thorax to the distal end of L3 at the wing apex (Gidaszewski, Baylac, & Klingenberg, 2009; Wallingford & Loeb, 2016). A second wing vein measurement was taken from the proximal end of the L4 longitudinal wing vein to the posterior crossvein and continued to the distal end of L4 at the wing apex (Leach, Stone, et al., 2019; Shearer et al., 2016). Thorax length was the distance between the anterior margin of the thorax (propleuron) and the posterior tip of the scutellum. Tibia length was measured as the distance between the distal end of the femur and the proximal end for the tarsus on the left foreleg. Color score assignments were made at the same time that body measurements were taken for each individual fly sample. A color score of 1–10, which indicated the percent melanization that was observed along the dorsal abdominal surface, was assigned to each of the five abdominal segments separately (Figure 1c; Shearer et al., 2016).

We also examined the effect of rearing temperature on color score assignments. All flies from this experiment were sourced from the colony in Geneva, NY. Five days after oviposition, 2‐3rd instar *D. suzukii* larvae were moved to one of four climate‐controlled growth chambers: (a) 25°C, 16L:8D; (b) 20°C, 12L:12D; (c) 15°C, 12L:12D; (d) 10°C, 12L:12D. Five days posteclosion, adults from each treatment were euthanized in 95% ethanol and stored at −4°C until evaluation. Color scores from each of the 5 abdominal segments were recorded in approximately 10 males and 10 females from each treatment group. In total, slighter fewer males were evaluated (*N* = 46 females; *N* = 34 males).

### 2.3 Winter morph development and survival

Approximately 100 mixed‐sex SM flies from NY laid eggs in diet bottles for 24 hr at 25°C (*N* = 3 bottles per treatment). To determine how WM trait development varies depending on the duration of time at cool temperatures, we manipulated the onset of chill by varying the timing at which we moved developing *D. suzukii* into a 15°C growth chamber, 12L:12D light cycle, 55% RH. There were 6 total development treatments: egg (24 hr after oviposition), 1st instar (48 hr after oviposition), 2nd instar (96 hr), 3rd instar (144 hr), and pupal stage (192 hr). We also included control flies that did not undergo a chill treatment (labeled “no chill”). We recorded the duration of development from oviposition to eclosion for each individual fly. In total, we collected data on development time over four replicates performed at different times during April, May, August, and September 2018.

Seventy‐two hours after eclosion, adult body size and abdominal color were compared among flies from each of the 6 chill duration treatments (egg: *N* = 42 Females: 14 Males; 1st instar: *N* = 25 F: 15 M; 2nd instar: *N* = 84 F: 35 M; 3rd instar: *N* = 52 F: 36 M; pupa: *N* = 50 F: 39 M; no chill: *N* = 29 F: 21 M). Body size was based on (a) wing length measured from the distal wing tip at wing vein L3 to the site of wing attachment on the thorax, (b) a second measure of wing length using the L4 wing vein, (c) thorax length measured from the anterior mesonotum to the posterior scutellum, and (d) tibia length. We measured each sample using a dissecting microscope set at 10× magnification. We then rated adult color based on percent of melanization present on the 3rd abdominal segment (Wallingford, Rice, Leskey, & Loeb, 2018).

To determine how exposure to cold temperatures at each life stage affects *D. suzukii* cold tolerance, we conducted additional laboratory‐based thermal stress test assays using the remaining flies not used for morphometric assessments. We evaluated survival in 10 treatments. In treatments, 1–6 flies were the same as those in the previous experiment (egg, 1st instar, 2nd instar, 3rd instar, pupa, and no chill). Four additional treatments allowed us to compare survival outcomes among cold tolerance larval‐exposed flies, with flies only exposed to cool temperatures as adults. Flies in treatments 7–8 were only subjected to 15°C early in adult maturation for the first 72 hr after eclosion (labeled “early adult”), or for 72 hr beginning when the adult flies were aged 1 week (labeled “late adult”). Treatments 9–10 were flies held at 15°C for 3 weeks after eclosion (labeled “Aged SM”) and WM flies (labeled “Aged WM”), respectively. This allowed us to measure the effect of long‐term cold exposure on cold tolerance, controlling for larval development conditions.

After each treatment was complete, we measured thermal susceptibility as the number of surviving flies after 72 hr at −5°C in a growth chamber (10L:14D; 25% RH; Kimura, 1988; Stockton et al., 2018). Five replicates (cohort bottles) were performed per life stage treatment. Each replicate comprised approximately 20 adult female flies contained in a standard *Drosophila* stock bottle. At the bottom of each bottle, 40 ml standard drosophila diet (previously described) was included to allow flies to feed *ad libidum*. The number of living and dead flies in each bottle was recorded after 24, 48, and 72 hr.

### 2.4 Regional variation in winter morph expression

In order to determine temporal and spatial variation in *D. suzukii* morphology, laboratories in New York (NY), Michigan (MI), Wisconsin (WI), Maine (ME), and Florida (FL) provided *D. suzukii* samples captured in baited wet traps (GL/SC‐5000‐12; Great Lakes IPM, Vestaburg, MI), using a four‐component olfactory SWD lure (GL/SC‐5100‐12; Great Lakes IPM). The lure was suspended from the interior lid of the trap, and the traps were filled with approximately 200 ml drowning solution (273 g table salt + 3.78 L water). The years from which samples were collected, and the months during which capture occurred, varied by site (Table 1). Mean daily temperature data were collected on site using data loggers to record outdoor ambient air temperature. The date at which the samples were recovered, as well as the mean weekly temperature 7 days prior to capture was recorded for all samples at each sampling site. After capture, all samples were stored in 95% ethanol and shipped to NY for evaluation. Samples were stored at −4°C until use. Measurements included L2 and L3 length, thorax length, tibia length and color score assessments of the 3rd abdominal segment, as previously described.

**TABLE 1:** Sampling site locations, dates of collection, *Drosophila suzukii* sample sizes, and mean monthly temperature data from each collection site

Sampling site	Year	Month	*N* =	Low °C^a^	Mean °C	High °C
Cornell University Geneva, NY	2017	September	20	15.41	20.79	27.67
		October	20	8.12	13.42	18.91
		November	20	−0.17	3.28	6.72
		December	20	−0.94	2.57	5.55
	2018	August	20	17.48	22.39	25.64
		September	41	14.59	18.78	22.28
		October	73	6.25	9.71	13.42
		November	50	0.18	3.53	6.69
Michigan State University East Lansing, MI	2016	August	10	20.00	24.81	29.35
		September	10	13.98	19.44	24.44
		October	10	11.67	16.48	21.02
		November	10	8.24	13.89	19.35
		December	10	−0.93	1.48	3.70
	2017	July	10	19.44	24.07	28.61
		August	10	15.83	21.67	27.04
		September	10	15.19	21.57	27.69
		October	10	13.43	20.74	27.78
		November	10	−2.41	2.87	7.78
		December	10	−0.74	5.09	10.65
University of Maine Orono, ME	2016	November	20	−0.97	3.75	8.15
	2018	September	71	12.67	17.62	22.68
		October	72	4.99	8.67	12.45
University of Wisconsin Madison, WI	2015	September	20	11.53	17.64	23.47
		October	20	5.65	10.97	16.11
		November	20	3.43	8.75	13.98
	2016	September	20	12.92	17.50	21.81
		October	20	6.90	11.94	16.76
		November	10	6.20	11.39	16.30
University of Florida Gainesville, FL	2017	December	31	6.19	13.35	20.27
		February	30	13.33	16.79	20.28
		March	20	14.68	18.24	21.90
	2018	January	84	2.53	9.66	16.41
		February	79	12.92	19.39	25.60
		March	79	6.84	15.18	23.22

^a^ Monthly average temperature near each sampling site.

### 2.5 Statistical analysis

#### 2.5.1 Defining winter morph traits

All reported analyses were performed in R i386 (version 3.6.1; the R Foundation for statistical computing (platform x86_64‐w64‐mingw32/x64); Vienna, Austria). We used multivariate analysis of variance (MANOVA; base R, no packages required) to determine how rearing temperature (25°C or 15°C) affected phenotypic expression of (a) fly size and (b) abdominal segment melanization. In model (a), we used wing, thorax, and tibia length as dependent variables to determine the effect of temperature on fly size. In model (b), we used the color scores from abdominal segments 1–5 as dependent variables. In both models, fly morphotype (SM or WM), sex, and source state (NY or NC) were included as independent variables. The dependent variable was assessed for individual flies, rather than aggregate groups. Mean melanization for each segment was referred to as a “color score.” Mean melanization across all segments was referred to as a “color rating.” Interactions between all three factors were included in the models and we did not use statistical blocking. We used Q‐Q plots of the residuals to determine if the assumptions of the models were met. Pillai's trace was used to estimate the effect size of each factor in both MANOVA models (Scheiner, 1993).

We used MANOVA to determine the effect of sex and rearing temperature on abdominal melanization, as described previously. For post hoc analysis, we ran a separate generalized linear model (GLM) for each abdominal segment and determined the effect of sex and temperature on color score. Pairwise differences between estimated marginal means were calculated using the R package “emmeans.”

#### 2.5.2 Winter morph development and survival

To assess the effect of chill duration on *D. suzukii* development time, we used a linear mixed model from the package “lme4” and the function *lmer.* Chill duration treatment was the fixed effect and replicate number (1–4) was the random effect due to variation in eclosion frequency. We used type 3 analysis of variance with Satterthwaite's method to determine goodness of fit using the package “car.” We then used the package “emmeans” for post hoc multiple mean comparisons among groups. We used separate linear mixed‐effects models for each body feature to determine the effect of chill duration and fly sex on L3, L4, thorax, and tibia size. Because color score data were not normally distributed, we used a GLMER with a Poisson distribution. Model outcomes are reported as Type II Wald chi‐squared tests. Post hoc comparisons among chill duration treatments were calculated using the package “emmeans” for each body feature.

To determine how the rearing/cold exposure treatments affected survival at cold temperatures, the time of death (24, 48, 72 hr, or no death (censored)) was recorded for each fly. We used Cox proportional hazards analysis from the R packages “survival” and “survminer” and the function *coxph* to model the effects of treatment (duration of developmental cold exposure) on survival probability. A mixed‐effects model was used to account for the random variation in survival among sample cohorts (replicate bottles; *N* = 5 per treatment). We used a log‐likelihood ratios test to determine the effect of treatment in our model. This is reported as analysis of deviance, chi‐squared values. Tukey's survival comparisons among treatments were conducted using the R package “emmeans” and were based on our Cox model. To compare survival at each time point, we used Fisher's multiple comparisons testing with the R package “RVAideMemoire” and the function *fisher.multcomp* due to occasional sparse cell size.

#### 2.5.3 Regional variation in morphotype expression

MANOVA (base R) was used to model the effects of mean weekly temperature, sampling site (ME, MI, NY, WI, and FL), and time of the year (month) on *D. suzukii* WM trait expression. The five morphometric characters were included in the model as the multivariate dependent factor (L3, L4, Thorax, Tibia, and abdominal color score). Pillai's trace values are reported.

We used GLMs to compare L4 length and color score at five distinct temperature brackets (below 5°C, 5–10°C, 10–15°C, 15–20°C, 20–25°C). We used the package “emmeans” for pairwise mean comparisons among sites within each temperature bracket.

We used linear regression to compare the relationship between temperature and various WM body traits from flies captured at each sampling site (Table 1). Some variables were combination factors of various trait sizes and color, yielding a new value that incorporated data from both traits. Because the relationship was stronger between L4 length and temperature, rather than overall wing size, L4 length was used for interpreting results. A new appearance factor, referred to as the “Appearance Score,” was generated by multiplying L4 length by abdominal color score for each sample.

## 3 RESULTS

### 3.1 Defining winter morph traits

**FIGURE 2:**
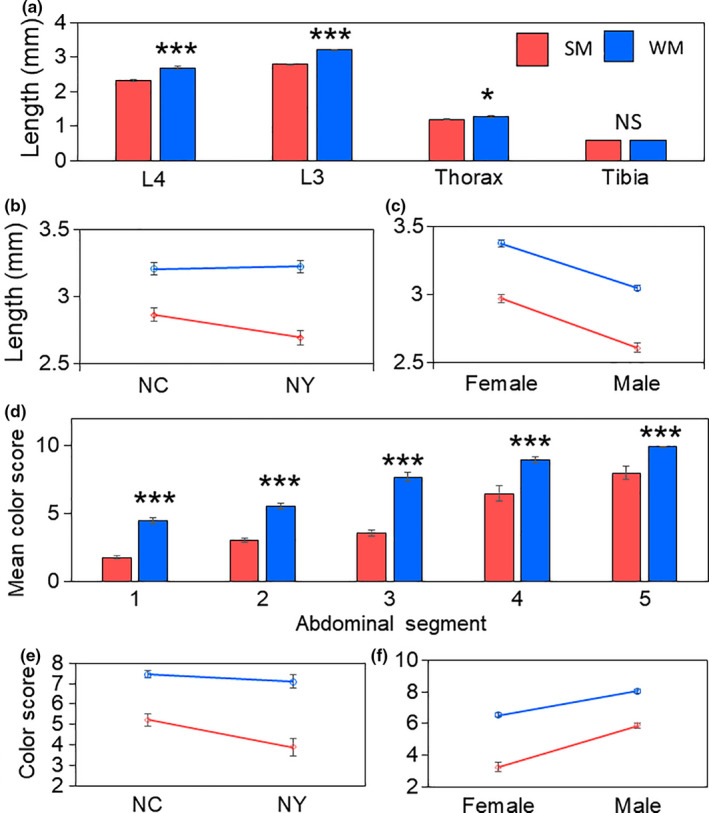
The association between *Drosophila suzukii* trait size and morphotype distinction (a‐c). Asterisks indicate statistically significant differences in mean (±*SEM*) trait size among SM (red) and WM (blue) flies (a). Differences in mean (±*SEM*) wing size among samples from NC and NY (b). Differences in mean (±*SEM*) wing size among female and male samples (c). The association between abdominal melanization and morphotype distinction (d‐f). Mean (±*SEM*) color scores among SM (red) and WM (blue) flies at each abdominal segment (d). Differences in mean (±*SEM*) color rating among samples from NC and NY (e). Differences in mean (±*SEM*) color rating among female and male samples (f)

Body size was differentially expressed in SM and WM flies, with WM flies being significantly larger than SM flies (Pillai = 0.81, *F* (1, 22) = 28.4, *p* < .001; Figure 2a; Table S1). There was no difference in size among flies from NY or NC (Pillai = 0.06, *F* (1, 22) = 9.50, *p* = .75; Figure 2b). However, females were significantly larger than males at both locations, and in both SM and WM flies. (Pillai = 0.77, *F* (1, 22) = 22.8, *p* < .001; Figure 2c). Wing size showed the greatest between‐group differences between WM and SM samples (Mean difference _L3_ = 0.42 ± 0.03; Mean difference _L4_ = 0.37 ± 0.06). While differences in thorax size were also significantly different among SM and WM flies (Mean difference _Thorax_ = 0.09 ± 0.01), the size of the difference was smaller than either measure of wing length. There was no difference in tibia size between the two morphotypes (Mean difference _Tibia_ = 0.00 ± 0.01.

Overall, the color scores associated with each abdominal segment were significantly higher in WM flies compared to SM flies (Pillai = 0.86, *F* (1, 64) = 72.74, *p* < .001; Figure 2d). Unexpectedly, SM samples reared in NC displayed higher color scores than SM flies from NY, despite similar rearing environments (Pillai = 0.44, *F* (1, 64) = 9.50, *p* < .001; Figure 2e). Color rating was significantly higher among male *D. suzukii* compared to females in both SM and WM flies (Pillai = 0.85, *F* (1, 64) = 69.15, *p* < .001; Figure 2f).

**FIGURE 3:**
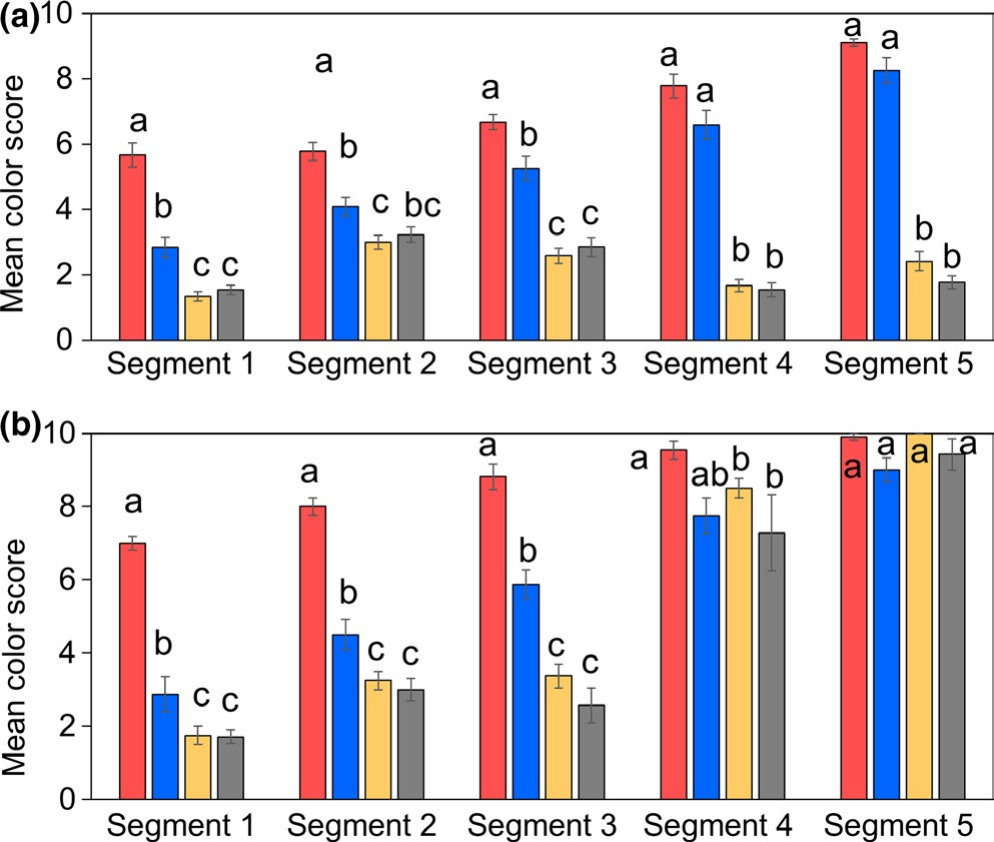
Mean (±*SEM*) color scores of female (a) and male *Drosophila suzukii* (b) on each abdominal segment (1–5). The development temperature is indicated by bar color: red = 10°C, blue = 15°C, yellow = 20°C, gray = 25°C. Significant differences among color score at the level of individual abdominal segments are indicated by different letters

In a separate experiment, we compared the abdominal melanization patterns of male and female *D. suzukii* reared at different temperatures. We found a predictable, negative relationship between abdominal melanization (higher scores are more melanized) and temperature (Pillai = 0.81, *F* (1, 76) = 59.57, *p* < .001); Figure 3, Figure S2). As previously observed, males were significantly darker than females (Pillai = 0.78, *F* (1, 76) = 50.51, *p* < .001); however, multiple comparisons tests showed that this effect was significant only for those flies reared at 10, 20, and 25°C, but not 15°C (Figure S3).

There was a significant interaction between sex and temperature, indicating that with decreasing temperature, abdominal darkening increased more in females than males (Pillai = 0.73, *F* (1, 76) = 39.82, *p* < .001). Furthermore, in females, there was a difference at each abdominal segment, while in males there were differences in abdominal segments 1–3, but not 4 and 5 as those segments were uniformly scored 10, or completely pigmented (Figure 3, Table S2). In males, the greatest differences between SM and WM flies occurred on the third abdominal segment (Mean difference = 5.11), while in females the greatest difference in color score occurred on the fourth abdominal segment (Mean difference = 4.55).

### 3.2 Winter morph development and survival

**FIGURE 4:**
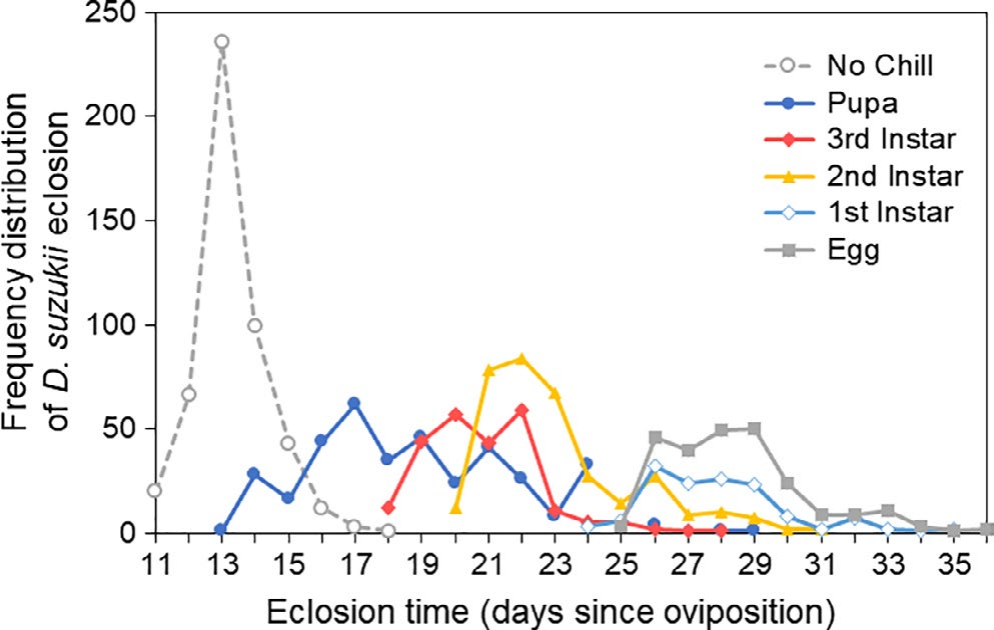
The effect of differential chill schedule on *Drosophila suzukii* development time and total eclosion among flies in the no chill control group (gray dashed), and those chilled beginning as pupae (dark blue), 3rd instar larvae (red), 2nd instar larvae (yellow), 1st instar larvae (light blue), or beginning as eggs 24 hr after oviposition (gray solid)

Larval development time increased as chill duration increased (*F* = 2,707.1, *df* = 5, 1,803.5, *p* < .001; Figure 4, Table S3). Development times increased by approximately 15 days in *D. suzukii* chilled during the egg stage (Mean = 28.23 ± 0.19 days), compared to flies never chilled (Mean = 13.07 ± 0.07 days). Pairwise comparisons showed significant differences in development time among each group except for “eggs” and “1st instar larvae,” although this was expected given the mean difference in development time between these two treatments was less than 1 day (Table 2).

**TABLE 2:** Development patterns of *Drosophila suzukii* reared at 15°C for differing durations beginning at each life stage

Life stage	Total eclosion^1^	Mean time^2^	*SD* ^3^	*SE* ^4^	CI^5^	Sig^6^
No chill	480	13.28	1.08	0.05	11.4–16.3	a
Pupa	370	18.80	3.04	0.16	16.6–21.5	b
3rd Instar	240	20.83	1.71	0.11	19.1–24.0	c
2nd Instar	339	23.05	2.24	0.12	21.2–26.1	d
1st Instar	136	27.89	2.15	0.18	26.2–31.1	e
Egg	247	28.47	2.14	0.14	26.6–31.5	e

^1^ Total number of insects that eclosed for each treatment.

^2^ The mean development time (days), from oviposition to the day of eclosion.

^3^ Standard deviation.

^4^ Standard error of the mean.

^5^ 95% confidence intervals.

^6^ Post hoc comparisons of estimated marginal means indicated by different letters (*α* = 0.05).

**FIGURE 5:**
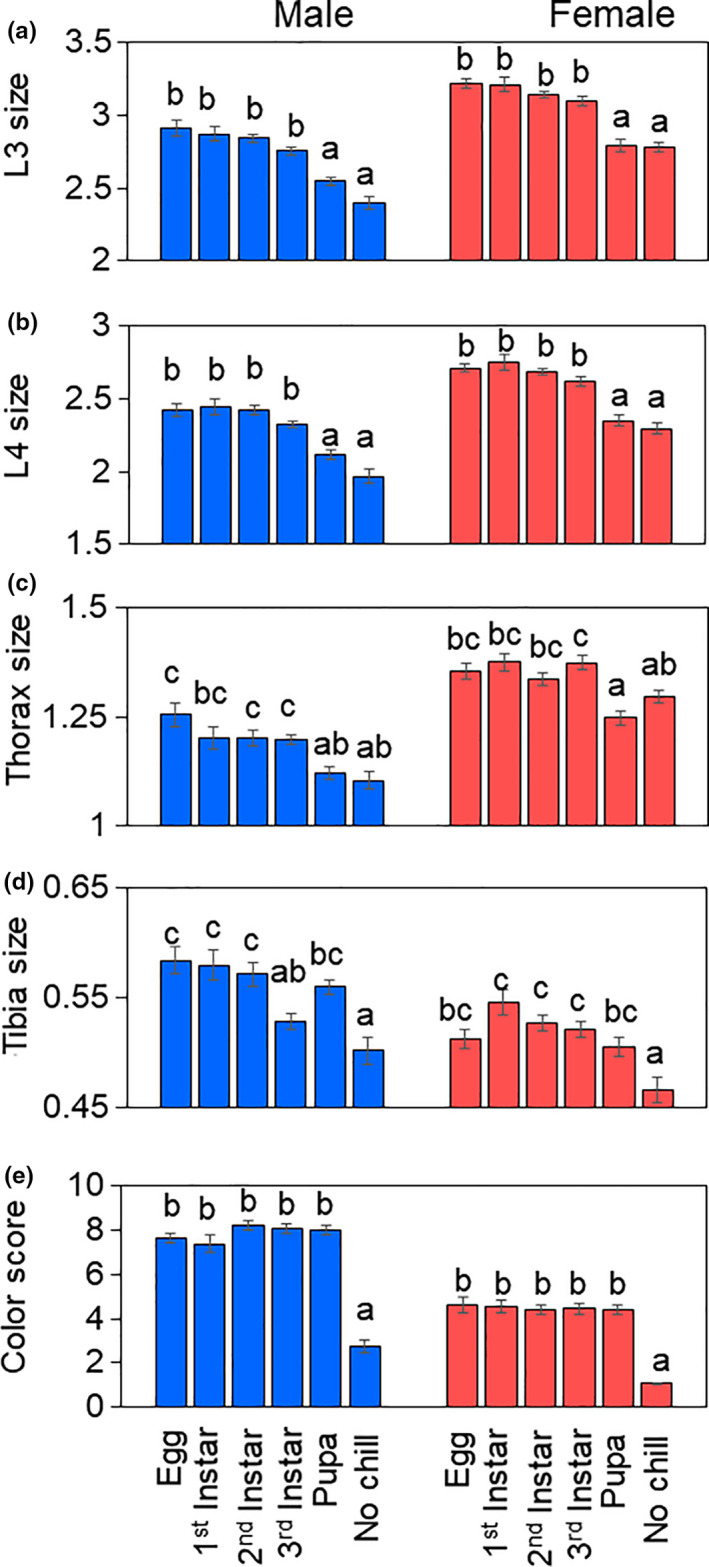
The effect of differential chill schedule on WM trait expression in male (blue) and female (red) *Drosophila suzukii*. Different letters indicate statistically significant differences in mean (± *SEM*) character size (a‐d) and mean (±*SEM*) color score (e)

Five days after eclosion, a subset of flies from each treatment group was used for morphometric evaluations. The timing and duration of exposure differentially affected the phenotypic expression of WM traits such that longer exposure to cool temperatures resulted in larger, but not darker flies (Figure 5). Increasing chill duration during larval development was associated with increased L3 length (χ^2^ = 509.67, *df* = 6, *p* < .001; Figure 5a), L4 length (χ^2^ = 497.52, *df* = 6, *p* < .001; Figure 5b), thorax length (χ^2^ = 105.29, *df* = 6, *p* < .001; Figure 5c), and tibia length (χ^2^ = 76.02, *df* = 6, *p* < .001; Figure 5d). Although wing, thorax, and tibia length was similar among all the larval chill treatments (“egg”, “1st instar”, “2nd instar,” “3rd instar”), flies that began the chill period during the pupal stage were generally smaller (Figure 5). The exception to this pattern occurred in tibia length, for which “3rd instar” tibia length was shorter than among those in the “pupa” group, although this difference was not significant (Figure 5d). While we observed differences in abdominal melanization among treatments (χ^2^ = 193.30, *df* = 6, *p* < .001; Figure 5e), this difference was only pronounced between treatment flies with varying durations of chill exposure and control flies that underwent no chill. All other larval and pupal treatment groups displayed similar levels of melanization regardless of chill duration.

**FIGURE 6:**
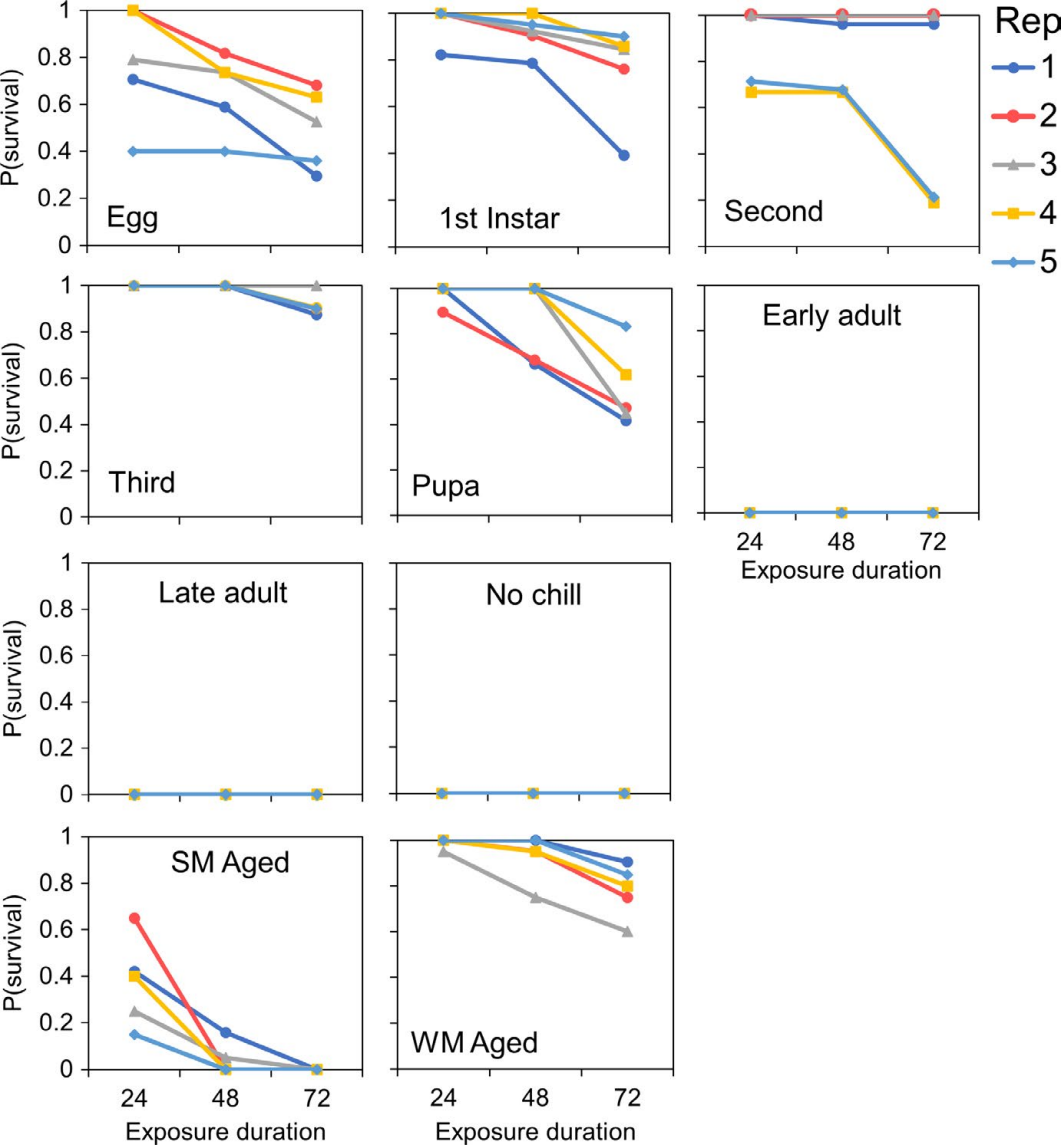
Proportional *Drosophila suzukii* survival during the stress test assay among treatment groups. Survival during exposure to −5°C was recorded for each fly at three predetermined time intervals (24, 48, and 72 hr). Replicate number (bottle number) is indicated by line color

The remaining flies were used to assess cold tolerance among flies reared at 15°C for varying durations. In total, we monitored the survival of 1,002 individual flies during the 72 hr exposure period in our thermal stress test (Figure 6). Our analysis showed that there was a significant treatment effect of developmental chill duration on adult survival outcomes (χ^2^ = 1,063.1, *df* = 9, *p* < .001; Table 3). Without pre‐exposure to 15°C during larval development, all flies died after 24 hr at our stress test temperature of −5°C (Figure 6). In contrast, all WM flies that experienced developmental chill at 15°C for durations ranging from 8–24 days showed improved survival relative to SM flies. Long‐term cold exposure in WM flies (“WM aged”) did not result in statistically significant increases in survival relative to other WM treatments; however, there was less within‐group variation. Among SM flies, while short‐term cold exposure that began during posteclosion was not associated with improved cold tolerance (“Early adult” and “Late adult”), long‐term exposure on otherwise SM flies (“SM aged”) at 15°C did result in improved survival, although this effect was only significant at 24 hr (Table S4).

**TABLE 3:** The effect of developmental chill duration on stress test survival when kept at −5°C for 72 hr

Fixed coefficients	*N*=^1^	Coef	Exp (Coef)	*SE* (Coef)	*Z*	*p*‐value	Sig^2^
Egg	102	−3.19	0.04	0.36	−8.72	<.001	b
First	96	−4.18	0.01	0.40	−10.46	<.001	ab
Second	112	−3.97	0.02	0.39	−10.25	<.001	ab
Third	96	−5.42	0.00	0.49	−10.98	<.001	a
Pupa	90	−3.56	0.03	0.37	−9.54	<.001	b
Posteclosion	99	0.00	0.99	0.36	0.00	1.00	c
Late adult	103	0.00	0.99	0.35	0.00	1.00	c
SM aged	99	−0.99	0.37	0.33	−2.98	.003	c
WM aged	102	−4.39	0.01	0.40	−10.89	<.001	ab
No chill (ref^3^)	103	‐	‐	‐	‐	‐	c

	Null	Integrated	Fitted
Log‐likelihood	−3,865.36	−3,333.83	−3,289.50

		St dev	Variance
Random effects	Replicate	0.485	0.235

^1^ The total number of insects tested in each treatment.

^2^ Tukey method of comparing survival proportions among treatment groups, based on our Cox mixed model.

^3^ Model reference value.

### 3.3 Regional variation in winter morph expression

When we compared WM trait expression of *D. suzukii* collected from different regions in the Eastern United States we found that there was a significant effect of sampling site (Pillai = 0.87, *F* (4, 20) = 51, *p* < .001) and month (Pillai = 0.61, *F* (8, 20) = 16, *p* < .001), but not mean weekly temperature (Pillai = 0.01, *F* (1, 5) = 2, *p* = .11; Table 4; Table S4). However, there were significant interactions between mean temperature and site (Pillai = 0.08, *F* (8, 40) = 4, *p* < .001), and mean temperature and month (Pillai = 0.21, *F* (7, 35) = 6, *p* < .001; Table S5). This is consistent with the hypothesis that seasonal changes in the phenology of *D. suzukii*, combined with regional differences in temperature, may produce predictable differences in the expression on morphological traits.

**TABLE 4:** The relationship between the expression of *Drosophila suzukii* body character size and abdominal color score and mean weekly temperature

Dependent variable(s)	*df*	*SE*	*F*	*p‐*value	*R^2^*
L3	38, 391	0.22	7.58	<.001	0.205
L4	38, 391	0.20	9.89	<.001	0.259
Thorax	38, 391	0.11	4.74	<.001	0.128
Tibia	38, 391	0.06	12.72	<.001	0.315
Color	38, 391	1.12	37.89	<.001	0.591
L3 * Color	38, 391	2.93	41.25	<.001	0.612
L4 * Color	38, 391	3.31	46.25	<.001	0.640
L3 * Thorax * Tibia * Color	38, 391	2.38	41.26	<.001	0.612
L4 * Thorax * Tibia * Color	38, 391	2.72	45.50	<.001	0.636

Note

Asterisks indicate variables calculated by multiplying the given dependent variables.

**FIGURE 7:**
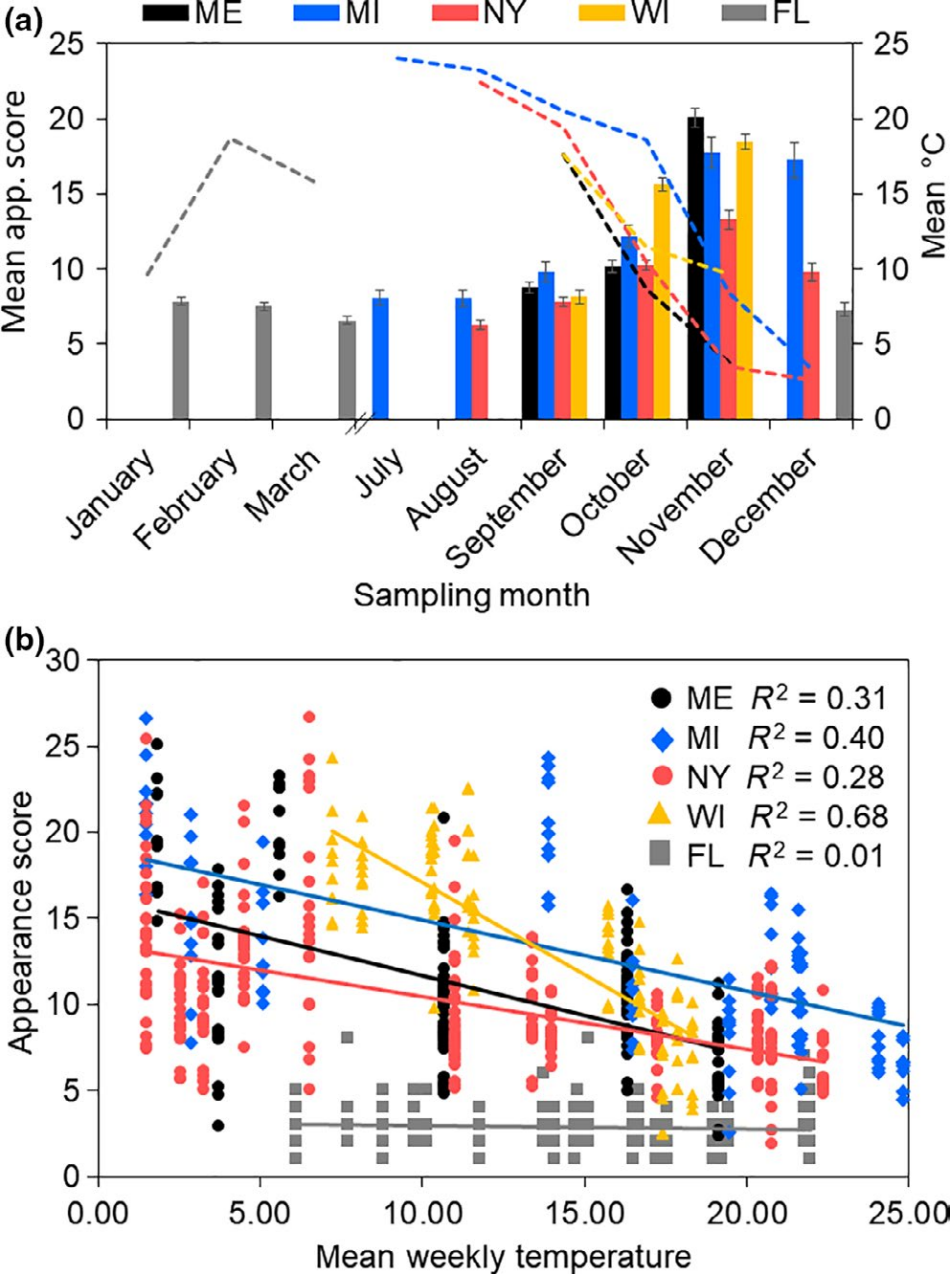
Mean WM trait expression (appearance = L4 × Color score; *Y*‐axis, left) in wild *Drosophila suzukii* captured throughout the year (a). Different line colors indicate different sampling site locations. Dashed lines indicate mean daily temperature throughout the year (*Y*‐axis, right). Each site initiated collections when *D. suzukii* became prevalent, during late summer. The relationship between *D. suzukii* appearance and mean daily temperature varied by sampling site (b). The effect size (adjusted *R* ^2^ values) of the relationship between appearance score and mean weekly temperature varied among sites

While overall temperatures decreased in NY, MI, ME, and WI each month from August–December, samples in some of these locations were larger and darker in color despite similar temperatures (Figure 7a,b, Tables S6 and S7). We observed that *D. suzukii* collected in ME were on average, larger than NY flies (Table S6), while in WI they were darker (Figure 7b; Table S7). We also found that the patterns of trait expression in FL did not follow the trends seen in the other states in which we sampled. *Drosophila suzukii* captured in FL were generally much smaller (Table S6) and lighter in color (Table S7) than samples collected from more northern locations.

In order to determine the strength of the relationship between each morphological trait and temperature, we compared the effect size of each trait using multiple regression models (Table 4). Among the individual traits recorded, color displayed the strongest relationship with mean weekly temperature (*R* ^2^ = 0.59). Each of the body size features was also significantly correlated with temperature, but the effect sizes (*R* ^2^ values) were smaller compared to color score (Table 4). Both wing measures displayed stronger correlations with temperature than tibia (*R* ^2^ = 0.32) or thorax size (*R* ^2^ = 0.13), likely indicating that these latter traits do not show large thermal variation. However, the effect size for the L4 wing vein (*R* ^2^ = 0.26) was larger than L3 (*R* ^2^ = 0.21), suggesting L4 would be a better predictor of abiotic conditions. A combined factor that is the product of L4 length and abdominal color score had the strongest relationship with temperature (*R* ^2^ = 0.64), indicating that this is the strongest abiotic predictor and best measure for characterizing flies as winter morph.

## 4 DISCUSSION

Among highly successful invasive species, the capacity to undergo adaptive changes in response to novel environmental stressors is considered one of the most significant indicators of their potential for ecological establishment (Agrawal, 2001; Chown, Slabber, McGeoch, Janion, & Leinaas, 2007; Davidson, Jennions, & Nicotra, 2011). However, to understand how an organism responds to environmental stress, it is critical to first determine which traits display the greatest phenotypic plasticity, and in doing so, define the criteria used for assessing morphotype variability. In the present study, we first attempted to identify the external traits that showed the greatest degree of variation between *D. suzukii* SM and WM morphotypes, yet displayed the smallest variation among individuals. Our data show that the reaction norm for larval development, and therefore morphotype trait expression, occurs along a continuum from 25°C to about 10°C, consistent with the niche temperature range observed in most *Drosophila* (Hoffmann et al., 2003). We observed, as have others, that as larval development temperature decreased, body size and abdominal melanization increased in a predictable manner (Leach, Stone, et al., 2019; Shearer et al., 2016; Wallingford & Loeb, 2016). Among flies reared in the laboratory under controlled environmental conditions, L3 wing vein length measuring greater than 3 mm, or L4 length greater than 2.5 mm was consistently associated with WM flies. While both wing vein measures were highly correlated, indicating that either would be appropriate to use, the strength of the relationship between wing length and temperature was stronger for the L4 measure, consistent with previously reported results (Leach, Stone, et al., 2019; Shearer et al., 2016). In addition to wing length, we also measured thorax and tibia length. Our initial experiments showed that although thorax length was significantly larger among WM flies, the difference was relatively small and showed the least between‐group variation between SM and WM flies. However, among the different chill duration treatments in experiment 2, these differences were more pronounced, and in our wild fly assessments thorax length was a highly significant factor. When we assessed tibia length, we found that although our initial experiments did not show large, consistent changes in tibia length due to rearing temperature, the multistate data revealed this as a strong predictor of seasonal change due to lower within‐group variation compared to other measures.

Abdominal color score was also significantly affected by changes in development temperature, although the results were less consistent than those using body size alone. This was because the degree of variation among individuals was quite high, despite consistency in differential expression between morphotypes. Our initial data indicated that a color score of 4 or greater (~40% melanization) on the 3rd abdominal segment of female *D. suzukii* is a conservative threshold value for WM identification. On average, females displayed a color score greater than 5 when reared at 15°C and less than 2 when reared above 20°C (Figure S3). Although all abdominal segments showed increased melanization with decreasing development temperature, segment 3 may be the most reliable for accurate WM assessment. This is for two reasons: First, we observed that segments 1 and 2 did not darken significantly above 10°C, indicating that these segments may not accurately reflect larval development temperature across the complete range of temperatures sufficient to induce WM traits. Second, among male flies segments 4–5 displayed elevated darkening above 20°C, consistent with what has been reported previously (Shearer et al., 2016). It is important to point out that the error associated with color score assignments was greater than for morphometric characters, indicating that this may be the most difficult WM trait to standardize. Indeed, color score is inherently more subjective than other measures and can be affected by lighting, the age of the samples, and observer perception, although we attempted to standardize each of these factors in our study and all samples were processed on the same equipment by the same observers, all in New York. Because we used a 10‐point scale in this study, it is unclear whether this has a benefit over the 5 point scale used in previous studies (Leach, Stone, et al., 2019; Shearer et al., 2016). Indeed, a smaller scale may result in less observer error, thereby reducing individual variation. Furthermore, it is likely that variation in color score can be attributed to additional abiotic factors such as photoperiod (Leach, Stone, et al., 2019; Shearer et al., 2016). While wing length does not appear to be significantly affected by short versus long day‐length independent of temperature (Leach, Stone, et al., 2019), photoperiod may have some effects on melanization. Indeed when flies are kept at relatively warm temperatures (>20°C), melanization decreased among flies kept on a shortened “winter” photoperiod compared to those on a longer “summer” photoperiod (Shearer et al., 2016). This suggests that the processes contributing to melanization are more complex than temperature alone (Ramniwas, Kajla, Dev, & Parkash, 2013). For that reason, abdominal color score may be a less reliable measure of WM trait expression than wing length.

It is also critical that beyond the external morphotype assignment given to species displaying seasonal polymorphic variation, we simultaneously understand the functional relevance of those external traits. The stress test assay revealed that while exposure to cool temperatures during larval development was critical to survival outcomes at temperatures below freezing, the timing and duration of that exposure was also a significant factor affecting morphotype expression and cold tolerance. Development time increased as exposure time increased, with egg and first instar treatments taking nearly 2 weeks longer to develop than SM flies. After eclosion, those WM adults were larger and darker in color than WM flies that began induction later in larval development, although this difference was not statistically significant. This indicates that the mechanism for external trait induction likely occurs quite late in larval development or even during pupation. Differences in trait expression and chill timing were more apparent beginning during the pupal stage, which was associated with smaller body size and decreased development time more similar to SM flies. However, the duration of cold exposure, even among those in the pupal treatment group, did not appear to affect cold tolerance. Rather, exposure to cool temperatures as juveniles, anytime from egg to pupa, was associated with increased survival. Interestingly, flies that began the chill window during the third instar stage were more cold tolerant than all other groups, while insects in the egg and 1st instar groups displayed poorer survival, which may have been caused by the stress of such an extended larval development period at cool temperatures. Although the age of the fly itself did not affect SM survival (all flies died within 24 hr), a 3‐week acclimation period did improve SM survival, although this was only significant during the first 24 hr. The same long‐term acclimation period in WM flies also appeared to improve survival and reduce within‐group variation. This is consistent with previous data suggesting the importance of adult acclimation in determining the cold tolerance of *D. suzukii* (Stockton, Brown, et al., 2019; Stockton et al., 2018; Stockton, Wallingford, et al., 2019; Wallingford & Loeb, 2016). These data indicate that internal physiological processes such as induced cold tolerance appear to be regulated by factors independent of, or at least in addition to, those regulating larval development and external morphology. For that reason, the functional significance of morphotype assignment may be more ambiguous that previously thought, at the very least, in response to short‐term cold stress. Unfortunately, there is surprisingly little literature available regarding the mechanisms regulating morphological shifts relative to cold hardening and acclimation, the latter of which have more extensively been investigated (Teets & Denlinger, 2013). Given the overlap of morphotypes observed in field populations (Guédot et al., 2018; Leach, Stone, et al., 2019), particularly during thermal transition periods in the fall, our data suggest that SM flies may be able to survive brief decreases in temperature below freezing. Future research should investigate whether SM flies continue to lay eggs after such events, as we would expect those offspring to be the primary overwintering population (Grassi et al., 2018; Rossi‐Stacconi et al., 2016).

The last question we investigated asked whether the threshold values for WM induction were consistent despite environmental variation in sampling location. To do this, we collected *D. suzukii* specimens from five locations in the United States (ME, NY, MI, WI, and FL) and observed spatial and temporal changes in WM trait expression in field‐collected specimens. We initially hypothesized that if morphotype variation was independent of mean temperature, it could indicate that different D. suzukii populations in the Eastern U.S. are genetically distinct (Ayrinhac et al., 2004; Hoffmann et al., 2003), despite data indicating these populations likely began from a single introduction in this part of the country (Fraimout et al., 2017). Indeed, wild flies from NY were generally smaller than those from ME, and lighter in color than those from MI, despite adjustments that allowed us to compare within similar temperature ranges. However, in our warmest sampling location (FL), although mean temperatures were theoretically sufficient to induce WM trait expression, few flies met the criteria for such categorization, suggesting that temperature‐independent environmental variation may be the likely cause of trait variation observed in our study (Chown, Jumbam, Sørensen, & Terblanche, 2009), which is more consistent with genetic data from allopatric populations of *D. suzukii* in its native Japan (Gotthard et al., 1995; Kimura, 2004). It is possible that in warmer climates, cool temperatures are often not stable enough below the threshold of 15°C to induce WM development characteristic of what we observe in the laboratory and in our Northern sites. Additionally, other abiotic factors, such as differential resource availability (Stockton, Brown, et al., 2019; Stockton, Wallingford, et al., 2019) and/or longer day length, may contribute to variation in external WM trait expression, accounting for the variation we observed among our various northern sites (Hoffmann et al., 2003; Hori & Kimura, 1998; Kimura, 2004). Although more research is needed to definitively determine the differential cause of the observed variation (genetic vs. environmental in origin), our data currently suggest that adaptive plasticity, as determined by differences in the abiotic environment, is the most likely driver of regional variation in this species. Given these results, it may be beneficial to employ regionally specific criteria for morphotype assignment that accounts for variation in WM trait expression.

Future research should address whether additional abiotic factors such as thermal stability and photoperiod may affect WM expression and cold tolerance thresholds (Leach, Stone, et al., 2019; Shearer et al., 2016). This is of economic and ecological importance due to the widespread effects of *D. suzukii* invasion. Furthermore, in the era of climate change there is concern that among invasive ectothermic species, phenotypic plasticity that favors adaptive responses to thermal variation may be the most significant factor predicting range expansion and total ecological impact (Bale & Hayward, 2010; Chown et al., 2007; Valladares et al., 2014). Although the present data indicate that at least external morphology varies among wild populations in the Eastern United States, it is unclear if the lower limits of survival are similar in Northern and Southern locations, as our laboratory data suggest. This could have significant implications for forecasting yearly infestation risk. Additionally, while the North American range of *D. suzukii* currently extends northward into Southern Canada, more research is needed to determine the additional behavioral and ecological mechanisms underlying survival among locally overwintered populations (e.g., availability‐dependent diet and refuge use) (Bal, Adams, & Grieshop, 2017; Stockton, Brown, et al., 2019; Stockton, Wallingford, et al., 2019; Tochen, Walton, & Lee, 2016; Wallingford et al., 2018). However, this remains a difficult problem to address because detection is difficult after mean daily temperatures drop below freezing (Rossi‐Stacconi et al., 2016; Stockton, Brown, et al., 2019; Stockton, Wallingford, et al., 2019). Since this species was first detected in the Northeastern United States and Great Lakes Region, *D. suzukii* has never been captured between mid‐January and May, when the population begins to re‐emerge (Bal et al., 2017; Guédot et al., 2018; Leach, Van Timmeren, et al., 2019). Although genetic analyses have just begun to address seasonal population stability in North American and Europe (Rota‐Stabelli et al., 2020), and there may be evidence of genetically stable pockets within these regions (J. Chiu, personal communication), these data are difficult to interpret given the amount of human‐directed movement of these pests, likely with interstate, and even international, fruit shipments. Ultimately, by continuing to study the morphological and genetic variation of various populations of *D. suzukii*, we may move toward a better, and even predictive, understanding of range expansion in this species, as well as other globally invasive arthropods.

## CONFLICT OF INTEREST

The authors have no conflicts of interest or competing interests relevant to this study or the science presented.

## AUTHOR CONTRIBUTIONS

**Dara G. Stockton:** Conceptualization (equal); data curation (lead); formal analysis (lead); investigation (lead); methodology (lead); project administration (lead); supervision (lead); validation (equal); visualization (equal); writing – original draft (lead); writing – review & editing (lead). **Anna K. Wallingford:** Conceptualization (lead); data curation (equal); investigation (equal); methodology (lead); project administration (equal); supervision (equal); visualization (equal); writing – original draft (equal); writing – review & editing (equal). **Gabrielle Brind'amore:** Conceptualization (equal); data curation (equal); investigation (equal); methodology (equal). **Lindsy E Iglesias:** Data curation (supporting); investigation (supporting); writing – review & editing (equal). **Lauren Diepenbrock:** Data curation (supporting); investigation (supporting); writing – review & editing (equal). **Hannah Burrack:** Conceptualization (supporting); data curation (supporting); funding acquisition (equal); investigation (supporting); methodology (supporting); project administration (equal); resources (equal); supervision (equal); writing – review & editing (equal). **Heather Leach:** Conceptualization (supporting); data curation (supporting); investigation (supporting); methodology (supporting); project administration (supporting); writing – review & editing (equal). **Elissa Ballman:** Data curation (supporting); investigation (supporting); project administration (supporting); writing – review & editing (equal). **Janet Van Zoeren:** Data curation (supporting); investigation (supporting); project administration (supporting); writing – review & editing (equal). **Rufus Isaacs:** Conceptualization (supporting); data curation (supporting); funding acquisition (equal); investigation (supporting); methodology (supporting); project administration (equal); resources (equal); supervision (equal); writing – review & editing (equal). **Oscar Liburd:** Conceptualization (supporting); data curation (supporting); funding acquisition (equal); investigation (supporting); project administration (equal); resources (equal); supervision (supporting); writing – review & editing (equal). **Francis Drummond:** Conceptualization (supporting); data curation (supporting); funding acquisition (equal); investigation (supporting); project administration (supporting); resources (equal); supervision (supporting); writing – review & editing (equal). **Christelle Guedot:** Conceptualization (equal); data curation (supporting); funding acquisition (equal); investigation (supporting); methodology (supporting); project administration (supporting); resources (equal); supervision (supporting); writing – review & editing (equal). **Greg M. Loeb:** Conceptualization (equal); data curation (equal); funding acquisition (equal); investigation (equal); methodology (equal); project administration (equal); resources (equal); supervision (equal); writing – original draft (equal); writing – review & editing (equal).

### Open Research Badges

This article has earned an Open Data Badge for making publicly available the digitally‐shareable data necessary to reproduce the reported results. The data is available at https://doi.org/10.5061/dryad.4j0zpc884.

## Supporting information

Supplementary Material

## Data Availability

The datasets generated and analyzed during the current study are publicly available in the data repository Dryad (https://doi.org/10.5061/dryad.4j0zpc884).
