# Supplementary material for: Seasonal polyphenism of spotted‐wing Drosophila is affected by variation in local abiotic conditions within its invaded range, likely influencing survival and regional population dynamics
Source: Ecol Evol. 2020 Jun 24;10(14):7669–85. doi: 10.1002/ece3.6491 (PMC7391339; doi:10.1002/ece3.6491)
Supplement: Supplementary file 1 — Supplementary Material [file ECE3-10-7669-s001.docx]

**Supplemental Figures**

**
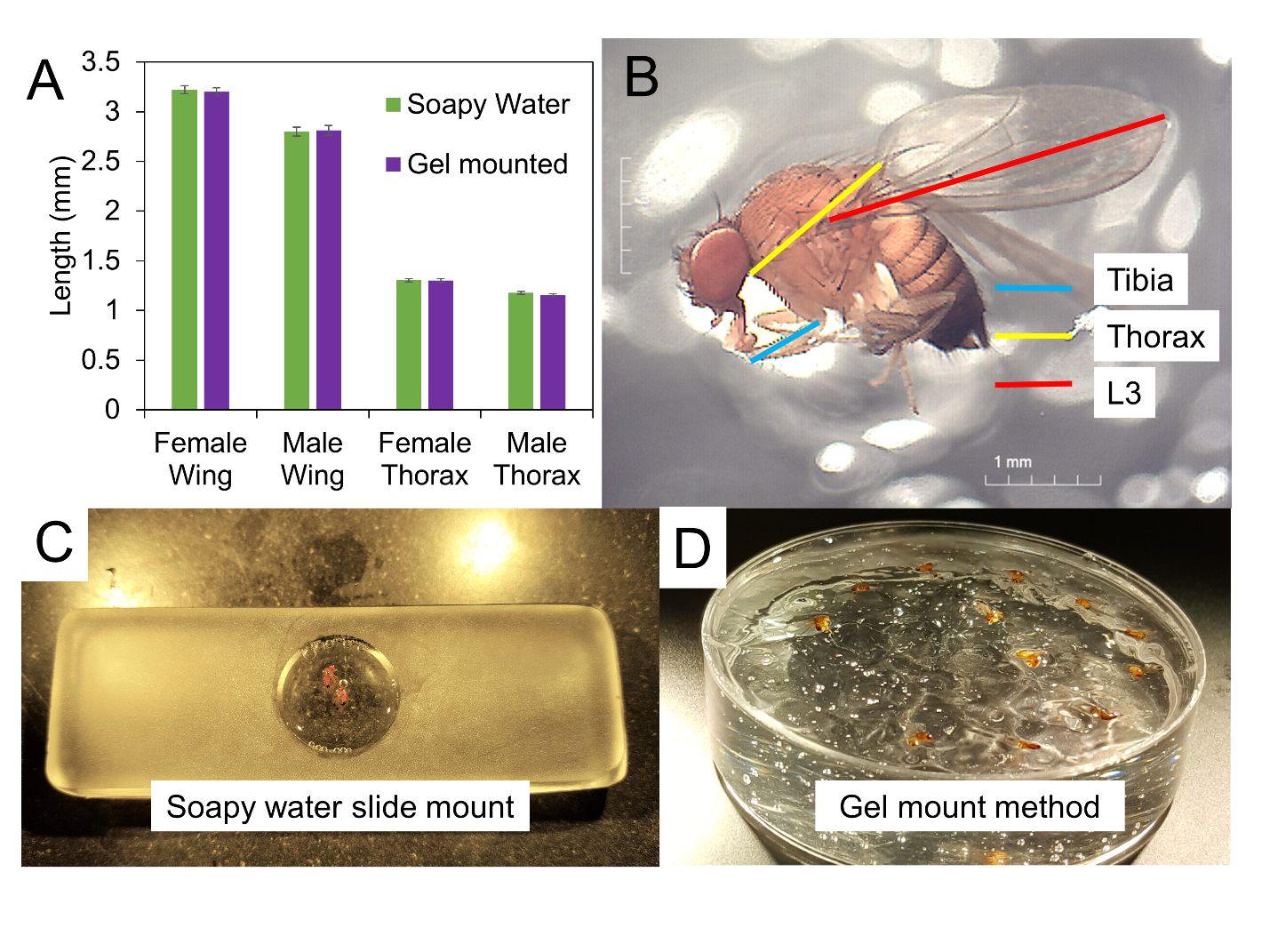
**

**Figure S1.** Preliminary data suggesting gel-mounted *D. suzukii* samples could be evaluated similarly to the conventional approach using a slide mount (A). The measures taken from each fly (N = 24) during this assay (B). Photograph of a conventional slide mount with the insect specimens suspended in soapy water (C). The alternative gel mount method showing multiple specimens arranged for efficient measurements in hand sanitizer gel within a plastic petri dish (D).

**
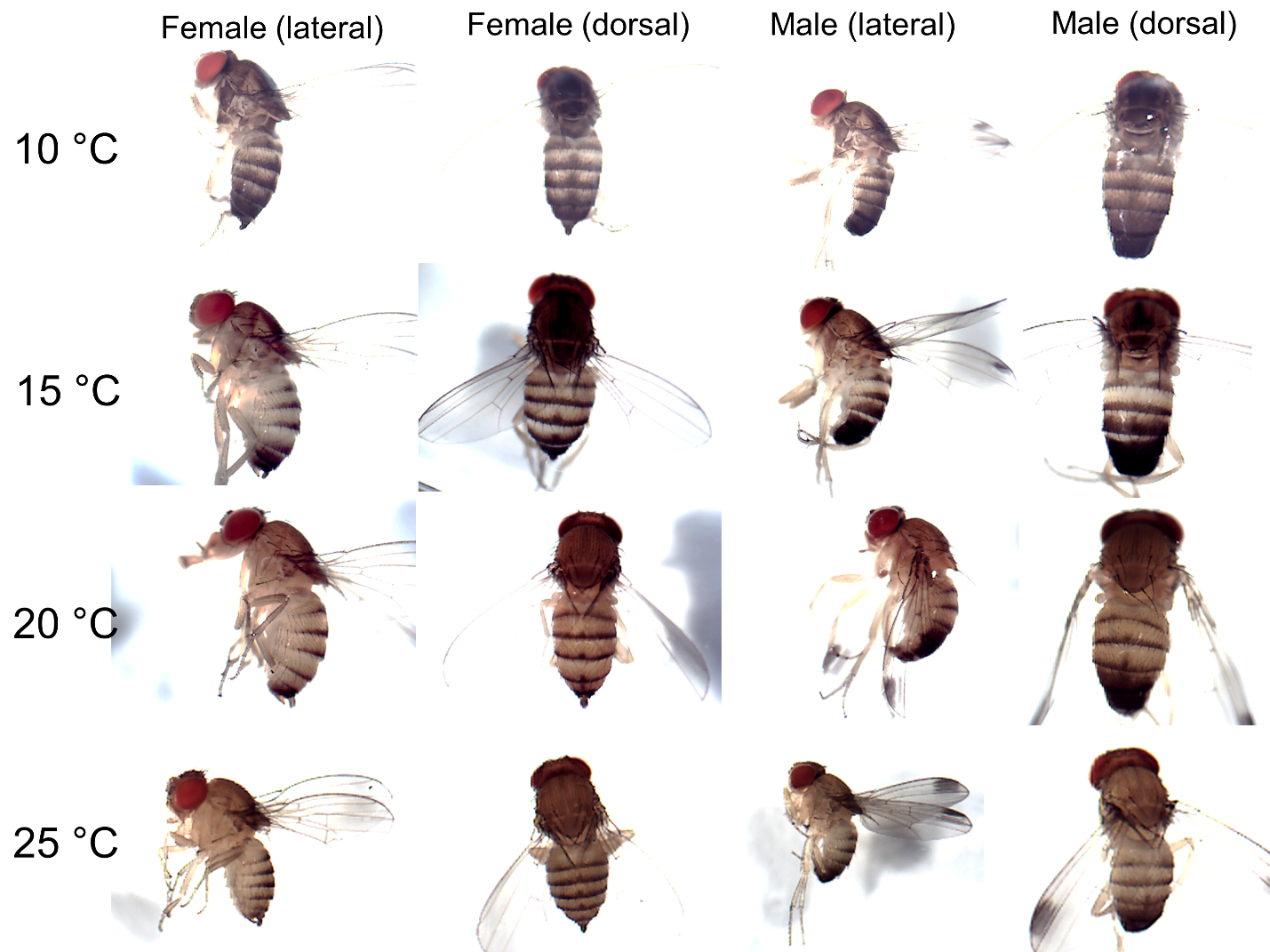
**

**Figure S2.** Representative images of the differences in abdominal color score among female and male *D. suzukii* reared at four different temperatures.


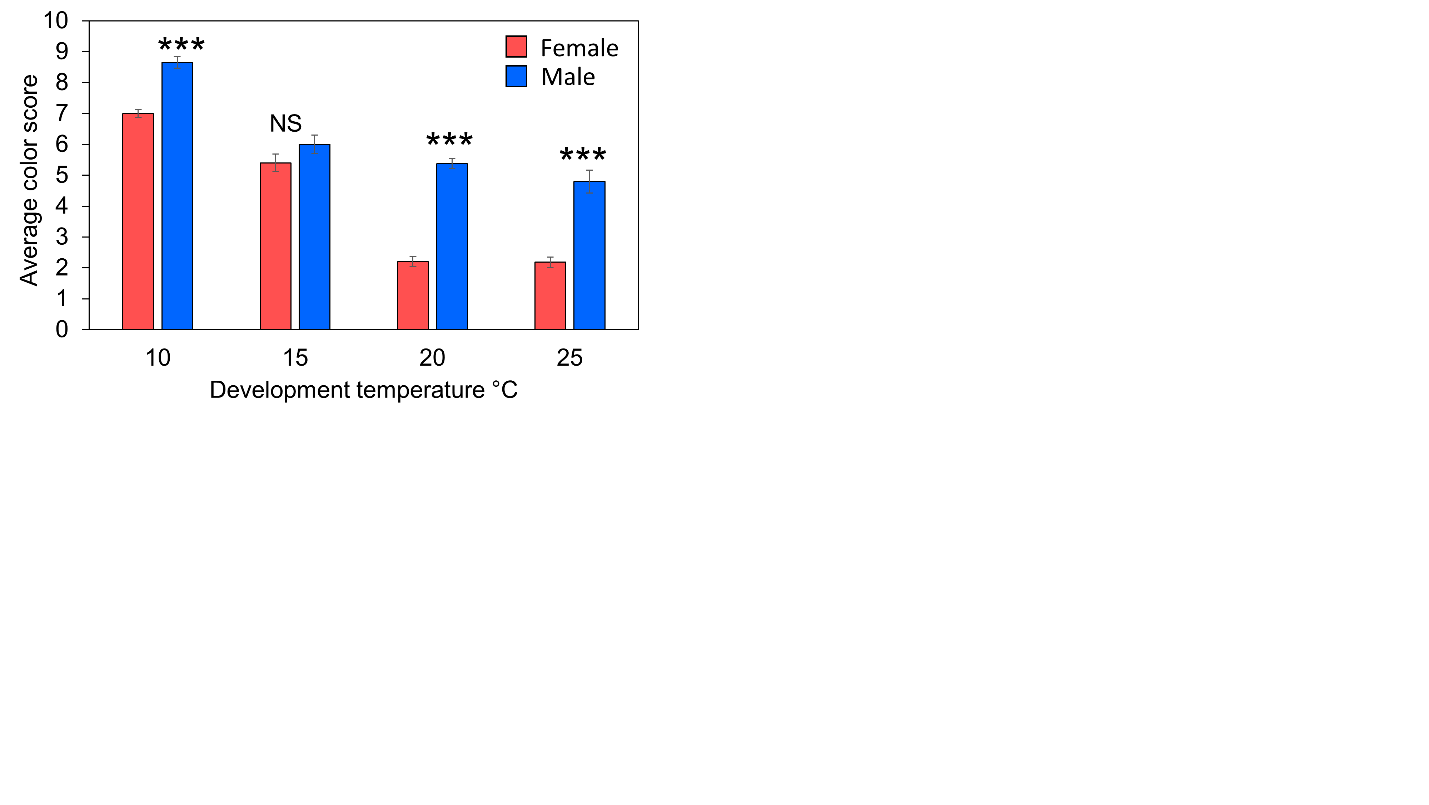


**Figure S3.** The effect of rearing temperature on *D. suzukii* abdominal melanization. Asterisks indicate statistically significant differences in mean (± SEM) color rating among male (blue) and female (red) flies; ***<0.001.

**Supplemental tables**

Table S1. MANOVA differences in body size (model 1) and abdominal color score (model 2) among flies in Experiment 1 based on morphotype (SM vs WM), fly sex, and colony source state (NY or NC).

| **Model** | **Model factor** | **Df** | **Pillai** | **F - value** | **P > F** |
| --- | --- | --- | --- | --- | --- |
| Model 1 | (Intercept) | 1 | 0.99 | 8788.1 | <0.001 |
| Body size | Morphotype | 1 | 0.81 | 28.4 | <0.001 |
|  | Sex | 1 | 0.77 | 22.8 | <0.001 |
|  | State | 1 | 0.06 | 0.4 | 0.751 |
|  | Morphotype x Sex | 1 | 0.04 | 0.3 | 0.859 |
|  | Morphotype x State | 1 | 0.30 | 2.9 | 0.060 |
|  | Sex x State | 1 | 0.12 | 0.9 | 0.458 |
|  | Morphotype x Sex x State | 1 | 0.16 | 1.3 | 0.295 |
|  | Residuals | 22 |  |  |  |
|  |  |  |  |  |  |
| Model 2 | (Intercept) | 1 | 0.99 | 1811.26 | <0.001 |
| Color Score | Morphotype | 1 | 0.86 | 72.74 | <0.001 |
|  | Sex | 1 | 0.85 | 69.15 | <0.001 |
|  | State | 1 | 0.44 | 9.50 | <0.001 |
|  | Morphotype x Sex | 1 | 0.79 | 46.76 | <0.001 |
|  | Morphotype x State | 1 | 0.38 | 7.55 | <0.001 |
|  | Sex x State | 1 | 0.47 | 10.72 | <0.001 |
|  | Morphotype x Sex x State | 1 | 0.44 | 9.55 | <0.001 |
|  | Residuals | 64 |  |  |  |

Table S2. The effect of *D. suzukii* sex and rearing temperature (10, 15, 20, 25 °C) on the color score of abdominal segments 1-5.

|  | **factor** | **df** | **F** | **P-values** |
| --- | --- | --- | --- | --- |
| Segment 1 |  | 79 |  |  |
|  | Sex | 1,78 | 31.69 | **<0.001** |
|  | Temperature | 3, 75 | 138.98 | **<0.001** |
|  | Sex * Temp | 3, 72 | 2.32 | 0.08 |
| Segment 2 |  | 79 |  |  |
|  | Sex | 1,78 | 33.59 | **<0.001** |
|  | Temperature | 3, 75 | 81.52 | **<0.001** |
|  | Sex * Temp | 3, 72 | 7.41 | **<0.001** |
| Segment 3 |  | 79 |  |  |
|  | Sex | 1,78 | 34.40 | **<0.001** |
|  | Temperature | 3, 75 | 96.92 | **<0.001** |
|  | Sex * Temp | 3, 72 | 4.27 | **0.008** |
| Segment 4 |  | 79 |  |  |
|  | Sex | 1,78 | 224.64 | **<0.001** |
|  | Temperature | 3, 75 | 54.52 | **<0.001** |
|  | Sex * Temp | 3, 72 | 23.81 | **<0.001** |
| Segment 5 |  | 79 |  |  |
|  | Sex | 1,78 | 550.55 | **<0.001** |
|  | Temperature | 3, 75 | 125.07 | **<0.001** |
|  | Sex * Temp | 3, 72 | 101.33 | **<0.001** |

Table S3. The effect of developmental chill duration on larval development times based on linear mixed model.

| **Fixed coefficients** | **Estimate ^a^** | **SE** | **df** | **t*-*value** | **P > t** |
| --- | --- | --- | --- | --- | --- |
| (Intercept) | 13.85 | 0.79 | 3.06 | 17.59 | <0.001 |
| Egg | 15.23 | 0.15 | 1803.06 | 99.86 | <0.001 |
| First | 14.78 | 0.19 | 1803.19 | 77.72 | <0.001 |
| Second | 9.81 | 0.14 | 1804.14 | 68.08 | <0.001 |
| Third | 7.66 | 0.16 | 1803.74 | 48.24 | <0.001 |
| Pupa | 5.18 | 0.14 | 1803.75 | 37.39 | <0.001 |
|  |  |  |  |  |  |
| Random effects | Variance | SD |  |  |  |
| Replicate | 2.43 | 1.56 |  |  |  |
| Residual | 3.76 | 1.94 |  |  |  |
| Number of observations: 1812, Replicates: 4 | | | | |  |

^a^ Estimates are relative to the “no chill” treatment group.

Table S4. Significant differences in survival among flies reared for different durations at 15 °C.

|  | **Total chill duration^1^** | **N=^2^** | **x̅ Surv^3^ 24** | **Sig.^4^** | **x̅ Surv 48** | **Sig.** | **x̅ Surv 72** | **Sig.** |
| --- | --- | --- | --- | --- | --- | --- | --- | --- |
| Egg | 24 | 102 | 0.762 | b | 0.647 | c | 0.5 | c |
| First instar | 22 | 96 | 0.948 | a | 0.896 | b | 0.708 | b |
| Second instar | 16 | 112 | 0.866 | ab | 0.848 | b | 0.643 | bc |
| Third instar | 12 | 96 | 1 | a | 1 | a | 0.917 | a |
| Pupa | 8 | 90 | 0.978 | a | 0.889 | b | 0.567 | bc |
| Post-eclosion | 3 | 99 | 0 | d | 0 | d | 0 | d |
| Late adult | 3 | 103 | 0 | d | 0 | d | 0 | d |
| No chill | 0 | 103 | 0 | d | 0 | d | 0 | d |
| SM aged | 21 | 99 | 0.374 | c | 0.04 | d | 0 | d |
| WM aged | 45 | 102 | 0.99 | a | 0.931 | ab | 0.782 | ab |

^1^ Total time the insects were chilled at 15 °C prior to testing including larval development and time post eclosion.

^2^ The total number of insects tested in each treatment.

^3^ Mean proportional survival at each time point.

^4^ Posthoc fisher’s multiple comparisons of sum survival proportions among treatment groups.

Table S5. MANOVA table showing the effects of collection site, month, and mean weekly temperature on *D. suzukii* appearance. The interactions are shown between all factors. Pillai values indicate effect size for each factor.

|  | **df** | **Pillai^1^** | **F** | **P*-*value** |
| --- | --- | --- | --- | --- |
| (Intercept) | 1 | 0.996 | 42480 | <0.001 |
| Site | 4 | 0.866 | 51 | <0.001 |
| Month | 8 | 0.614 | 16 | <0.001 |
| Temperature | 1 | 0.010 | 2 | 0.106 |
| Site x Month | 8 | 0.395 | 10 | <0.001 |
| Site x Temperature | 4 | 0.078 | 4 | <0.001 |
| Month x Temperature | 7 | 0.213 | 6 | <0.001 |
| Site: Month x Temperature | 6 | 0.068 | 2 | <0.001 |
| Residuals | 931 |  |  |  |

^1^Pillai’s trace (0-1) with increasing values indicative of greater effect size

Table S6. Variation in L4 length among sites depending on temperature.

| **Site** | **Below 5 °C** | **5 - 10 °C** | **10 - 15 °C** | **15 - 20 °C** | **20 - 15 °C** |
| --- | --- | --- | --- | --- | --- |
| FL | - | 2.45^1^ ± 0.03^2^ (64)^3^ a^4^ | 2.56 ± 0.02 (99) a | 2.55 ± 0.02 (120) a | 2.62 ± 0.04 (40) b |
| ME | 2.70 ± 0.03 (31) a | 2.75 ± 0.04 (10) b | 2.69 ± 0.02 (51) b | 2.62 ± 0.02 (71) c | - |
| MI | 2.65 ± 0.04 (20) a | 2.64 ± 0.05 (10) ab | - | 2.51 ± 0.05 (30) abc | 2.38 ± 0.03 (50) a |
| NY | 2.74 ± 0.02 (94) a | 2.60 ± 0.05 (20) ab | 2.57 ± 0.03 (69) a | 2.37 ± 0.05 (21) a | 2.49 ± 0.03 (60) a |
| WI | - | 2.50 ± 0.03 (20) a | 2.50 ± 0.02 (40) ab | 2.46 ± 0.03 (50) ab | - |
|  |  |  |  |  |  |
|  |  | **df** | **Deviance** | ***F* - value** | ***P* > *F*** |
| Temperature Bracket | | 4, 965 | 5.06 | 27.47 | <0.001 |
| Site | | 4, 961 | 1.63 | 8.86 | <0.001 |
| Bracket x Site | | 11, 950 | 3.08 | 6.07 | <0.001 |

^1^ Mean L4 length at each site within the temperature range indicated above.

^2^ Standard error of the mean

^3^ Sample size of the given estimates (n = x)

^4^ Posthoc comparisons in mean L4 length among sites within each temperature bracket (α = 0.05)

Table S7. Variation in abdominal color score among sites depending on temperature.

| **Site** | **Below 5 °C** | **5 - 10 °C** | **10 - 15 °C** | **15 - 20 °C** | **20 - 15 °C** |
| --- | --- | --- | --- | --- | --- |
| FL | - | 3.25^1^ ± 0.14^2^ (64)^3^ a^4^ | 2.79 ± 0.10 (99) a | 2.67 ± 0.09 (120) a | 3.20 ± 0.22 (40) a |
| ME | 5.13 ± 0.38 (31) a | 7.40 ± 0.31 (10) c | 3.63 ± 0.15 (51) b | 3.34 ± 0.13 (71) b | - |
| MI | 6.80 ± 0.34 (20) b | 5.00 ± 0.33 (10) b | - | 5.10 ± 0.36 (30) c | 4.10 ± 0.17 (50) b |
| NY | 4.46 ± 0.16 (94) a | 5.80 ± 0.46 (20) b | 3.57 ± 0.14 (69) b | 3.33 ± 0.16 (21) ab | 2.95 ± 0.13 (60) a |
| WI | - | 7.10 ± 0.19 (20) c | 7.27 ± 0.16 (40) c | 3.74 ± 0.19 (50) b | - |
|  |  |  |  |  |  |
|  |  | **df** | **Deviance** | ***F* - value** | ***P* > *F*** |
| Temperature Bracket | | 4, 965 | 389.24 | 62.89 | <0.001 |
| Site | | 4, 961 | 712.85 | 115.17 | <0.001 |
| Bracket x Site | | 11, 950 | 333.05 | 19.57 | <0.001 |

^1^ Mean color score at each site within the temperature range indicated above.

^2^ Standard error of the mean

^3^ Sample size of the given estimates (n = x)

^4^ Posthoc comparisons in mean color score among sites within each temperature bracket (α = 0.05)
